# A Qualitative Analysis of the Experiences of People Who Resumed Smoking Following Exclusive Electronic Nicotine Delivery Systems Use

**DOI:** 10.1093/ntr/ntac157

**Published:** 2022-11-30

**Authors:** Lindsay Robertson, Kealey-Rei Sanford, Anaru Waa, Janet Hoek

**Affiliations:** Department of Preventive and Social Medicine, University of Otago, Dunedin, New Zealand; Department of Public Health, University of Otago, Wellington, New Zealand; Department of Public Health, University of Otago, Wellington, New Zealand; Department of Public Health, University of Otago, Wellington, New Zealand

## Abstract

**Introduction:**

For electronic nicotine delivery systems (ENDS) to reduce harms caused by smoking, people who smoke must be able to switch to exclusive ENDS use without subsequently returning to smoking. Identifying factors prompting a return to smoking among former exclusive ENDS users is crucial, yet few qualitative studies have probed experiences of this process.

**Aims and Methods:**

We conducted in-depth, semi-structured interviews with 20 people (seven indigenous Māori and 13 non-Māori) who smoked tobacco at least weekly, had smoked at least 100 cigarettes in their lifetime, and reported using ENDS to stop smoking cigarettes for at least 30 days (ideally, within the preceding 6 months). We explored their experiences of ENDS use, probed critical return-to-smoking settings and triggers, and analyzed strategies that could promote sustained smoking abstinence. We managed data using *NVivo12* and used a reflexive thematic analysis approach to interpret the transcripts.

**Results:**

We identified three themes that explained participants’ experiences. ENDS performed a *functional role* by mimicking some aspects of smoking. Yet participants experienced ENDS as *inauthentic* and unsatisfying across physical, social, and affectual domains, including in the most common return-to-smoking situations. Furthermore, fewer constraints on ENDS usage led participants to feel they could *perpetuate addiction and risk of harm*.

**Conclusions:**

Return to smoking reflected two factors: ENDS’ failure to replicate core smoking attributes that remained appealing, and the burden of self-regulation required when using ENDS. Understanding and informing people about the challenges involved in transitioning to ENDS, beyond obtaining sufficient nicotine, could help support informed ENDS use and may potentially prevent people returning to smoking.

**Implications:**

Our study extends our understanding of the satisfaction people seek when attempting to transition from smoking to exclusive ENDS use, and how ENDS’ failure to replicate that satisfaction, in addition to uncertainty about ENDS-related risks, contributes to smoking resumption. Satisfaction went beyond nicotine delivery, and included affective experiences, maintenance of rituals, rewards, and social connections. Conceptualizing satisfaction more broadly could support a richer understanding of factors that prompt return to smoking. People might manage challenges more effectively if they understood these before attempting to switch from smoking to ENDS, and if they are advised to monitor and regulate their ENDS use. Educational resources and behavioral support could provide more guidance on these points.

## Introduction

Most people who try to quit smoking unaided will relapse within the first week of their cessation attempt and only 3% to 5% remain abstinent 6 to 12 months after quitting.^1^ Contributors to smoking relapse among recent quitters include physiological and emotional withdrawal (e.g., low mood, irritability, anxiety, changes in sleep quality, appetite, and heart rate),^2,3^ smoking urges,^2,4^ low self-efficacy,^5^ stress,^6,7^ loss of pleasure associated with smoking,^7^ and alcohol consumption.^2^ Desire to recover a residual “smoker” social identity may also cue relapse,^8–10^ as may being among people who smoke or in settings where cigarettes are easily available.^2,7,11^

High relapse rates, even among people motivated to quit, have led many to try switching to electronic nicotine delivery systems (ENDS).^12^ ENDS deliver nicotine efficiently and mimic some behavioral aspects of smoking, and appear effective at helping some people stop smoking.^13^ A systematic review concluded that for every 100 people using nicotine-containing ENDS to stop smoking, approximately 9 to 14 people would stop successfully, compared with approximately 6 of 100 people using nicotine replacement therapy.^13^ Given that cessation support can protect against relapse,^3^ ENDS use could theoretically reduce the risk of smoking resumption.^4,14,15^ A cohort study supports this reasoning as former smokers who used ENDS reported greater confidence in remaining smoke-free compared to non-ENDS users.^16^ However, two recent meta-analyses found ENDS use was associated with an increased risk of smoking resumption.^17,18^ These varied findings raise questions over the role of ENDS in supporting smoking abstinence.

As noted above, studies have identified contributors to smoking resumption amongst recent quitters;(e.g., 5, 6) others have suggested that dual use of ENDS and cigarettes results from efforts to manage tobacco control measures and comply with social norms.^19^ Very few studies have conducted in-depth analyses of contributors to smoking resumption following exclusive ENDS use or explored how those experiences compare to other cessation attempts. Understanding the factors that can lead to smoking resumption among former exclusive ENDS users is crucial if ENDS are to improve population health and reduce long-standing inequities resulting from tobacco use. We explored the experiences of New Zealanders (both Māori and non-Māori) who were formerly exclusive ENDS users and who had resumed smoking. We aimed to examine common cues, settings and triggers to smoking resumption, and identify strategies that could promote sustained smoking abstinence.

## Methods

### Sample and Recruitment

We used participants aged 18 years and over from Dunedin and Wellington using social media and community advertising, and by drawing on whanaungatanga (kinship) networks to use additional Māori participants. Eligible participants had smoked at least 100 cigarettes in their lifetime and currently smoked at least weekly, all reported using ENDS to stop smoking cigarettes for at least 30 days (ideally, within the preceding 6 months). Participants who experienced up to five brief smoking lapses (i.e., one-off cigarettes or puffs) during the 30-day abstinence period were eligible; current ENDS use did not affect eligibility.

Using a brief online screening survey, we ascertained potential participants’ eligibility; our purposeful sampling approach stratified potential participants by ethnicity, gender, age, and current smoking (daily versus weekly) and enabled us to achieve broad demographic representation.^20^ All participants received a $40 NZD gift voucher to recognize costs incurred while participating in the research. A delegated authority from the Department of Public Health reviewed and approved the research, which the University of Otago Human Ethics Committee (ref D20/199) subsequently confirmed. We engaged Māori members and advisors within our research team (AW) and from Hāpai te Hauora (a Māori health advocacy service), who provided guidance on the study design and protocol. We collaborated with KS (a Māori research internee) to collect some study data, and sought further input and feedback on themes from Hāpai te Hauora researchers, to ensure Māori voices were included in all phases of the study.

### Data Collection

Our semi-structured in-depth interview guide (Supplementary File 1) explored participants’ current and past smoking, and probed motivations for ENDS uptake. Participants discussed ENDS initiation and transition to exclusive ENDS use, their lapses and smoking resumption (i.e., context, contributors, consequences), and their future intentions regarding smoking and ENDS use. We used core questions and probes but retained flexibility in questioning so participants directed the discussion. All participants provided written informed consent before commencing, and interviews lasted on average 53 minutes (range: 37 to 63 minutes). LR, JH, and KS undertook the interviews, which continued until ongoing transcript reviews confirmed data redundancy (i.e., no new idea elements in two consecutive interviews).^21^

### Data Analysis

We recorded interviews, which were transcribed verbatim by an online service (rev.com), and checked transcripts for accuracy. We interpreted the data using an inductive reflexive thematic analysis approach, where we read and reread transcripts, reflected on our interactions with participants, and developed, reviewed and refined themes.^22–24^ LR and JH initially coded three transcripts independently using a line-by-line open-coding approach, then met to discuss and compare interpretations, and agree on preliminary codes. LR and JH then used these to code a further two transcripts before conferring to review and refine these, and discuss initial themes. LR coded the remaining transcripts, adding new codes as appropriate; she and JH met frequently to review codes and discuss themes that could represent these. As KS was no longer available, a Hāpai te Hauora researcher independently reviewed three transcripts from Māori participants to offer a Te Ao Māori perspective and help validate the themes and sub-themes developed. Our similar interpretations supported presenting the findings from the total sample. We managed the data using NVivo12.

When interpreting the data, we used a social constructionist epistemology that aligned with our interest in participants’ lived experiences of exclusive ENDS use before resuming smoking. We reflected on our own roles as current nonsmokers and nonvapers; as health researchers with experience of interviewing people who smoke and use ENDS, we brought pre-existing research knowledge to this study but our personal histories of tobacco and ENDS use differed from those of our participants.^25^ To maintain sensitivity to the lead researchers’ identity as non-Māori, we worked alongside Māori researchers to formulate the research questions and study design, and during data collection and interpretation. When presenting quotations, we removed filler words (i.e., “um”, “like” etc.) without replacement ellipses to improve readability.^26^ Because earlier studies have documented descriptive aspects of ENDS users’ experiences, preferences, and challenges (e.g., flavor and smell, affordability, ease or convenience, device maintenance, breakage potential, health concerns), e.g., ^27, 28^ we focused on participants’ attempt(s) to switch to ENDS, the proximal factors that led to smoking resumption, and comparisons to any past cessation attempts.

## Results

### Participants’ Characteristics

The sample comprised 11 women and nine men (age range: 20–58 years; median: 28 years). Twelve participants were New Zealand European, seven identified as Māori, and one as “Other” (i.e., European). Thirteen smoked daily and six at least weekly. While all participants met our eligibility criteria, we identified five as “accidental quitters” and the remaining 15 as “deliberate quitters”.^15^Table 1 outlines participants’ characteristics and their smoking and ENDS-use behaviors.

**Table 1.** Participant Characteristics

Pseudonym	Sex	Age	Ethnicity	Accidental or deliberate quitter	Current no.of cigarettes per day (or per week)	Time to first cigarette after waking	Years since weekly smoking began	What type of cigarettes do you smoke?	Current ENDS use
Anita	F	21	NZ European	Accidental	3 per day	> 60 min	3 years	RYO and tailor-made cigarettes	Daily
Callum	M	36	NZ European	Accidental	7 per day	< 5 min	21 years	Only RYO	Daily
Corinne	F	24	NZ European	Deliberate	8 per day	6–30 min	8 years	Only RYO	No longer uses ENDS
Erin	F	22	NZ European	Deliberate	3 per day	6–30 min	1 year	Only RYO	Daily
Hamish^ǂ^	M	21	NZ European	Accidental	20 per week	> 60 min	4 years	RYO and tailor-made cigarettes	No longer uses ENDS
Heather	F	58	NZ European	Deliberate	15 per day	< 5 min	40 years	RYO and tailor-made cigarettes	Daily
Hugh^ǂ^	M	28	Other (European)	Deliberate	15 per week	> 60 min	8 years	Only tailor-made cigarettes	No longer uses ENDS
Isaac	M	31	NZ European	Deliberate	2 per day	< 5 min	7 years	Only tailor-made cigarettes	Daily
Jake	M	21	NZ European	Accidental	1 per day	> 60 min	2 years	Only RYO	Daily
Jeff	M	27	Māori/ NZ European	Deliberate	10 per day	< 5 min	8 years	RYO and tailor-made cigarettes	At least weekly
Leigh^ǂ^	F	26	Māori/ NZ European	Deliberate	20 per week	6–30 min	8 years	Only tailor-made cigarettes	Daily
MacKenzie	F	20	Māori/ NZ European	Deliberate	7 per day	6–30 min	8 years	RYO and tailor-made cigarettes	No longer uses ENDS
Mikaere	M	25	Māori/ NZ European	Deliberate	15–20 per day^*^	^**^	11 years	Only RYO	Daily
Natalie	F	32	NZ European	Deliberate	10 per day	31–60 min	14 years	Only tailor-made cigarettes	Daily
Pippa^ǂ^	F	36	NZ European	Deliberate	2 per week	6–30 min	21 years	Only tailor-made cigarettes	Daily
Sam	M	31	NZ European/ Other	Deliberate	8 per day	> 60 min	13 years	Only RYO	Daily
Sarah^ǂ^	F	21	Māori/ NZ European	Accidental	3 per week	> 60 min	3 years	Only tailor-made cigarettes	Daily
Stacey^ǂ^	F	41	Māori/ NZ European	Deliberate	60 per week	< 5 min	28 years	Only RYO	Daily
Tabitha	F	46	NZ European	Deliberate	15 per day	6–30 min	31 years	RYO and tailor-made cigarettes	Daily
Warren	M	41	Māori/ NZ European	Deliberate	10 per day	< 5 min	29 years	Only tailor-made cigarettes	No longer uses ENDS

^ǂ^Indicates participants reported smoking at least weekly (but not daily) at the time of interview; all other participants reported smoking daily.

^*^Estimated based on consuming one packet of RYO every 3 to 4 days

^**^data for this question were not recorded.

### Motivation for Transitioning to ENDS

Accidental quitters’ ENDS uptake did not reflect a purposeful effort to reduce or stop smoking cigarettes, but resulted from device sharing or gifting (though one participant reported buying an ENDS for its novelty). By contrast, deliberate quitters initiated ENDS use in an explicit attempt to reduce or stop cigarette smoking, most commonly motivated by the high financial cost of tobacco in New Zealand. Aside from health concerns, the other main reason for wanting to reduce tobacco use was the perceived unacceptability or stigma of smoking; participants sought to eschew both a “smoker” identity and the tainting smell of tobacco smoke.

### Thematic Analysis

We identified three themes that explained participants’ experiences of resuming smoking after exclusive ENDS use. First, ENDS performed a positive *functional role* by mimicking aspects of smoking and offering some appealing experiences that several participants preferred over nicotine replacement therapies (NRT). Yet despite this functionality, some participants experienced ENDS as *inauthentic* across physical, social, and affectual domains, including in the most common return-to-smoking settings. Third, since ENDS had fewer constraints (e.g., fewer areas where usage was restricted or perceived as such), many participants reported sustained ENDS consumption, which they felt could *perpetuate addiction and risk of harm* and required self-regulation—an additional effort above that required to resist cigarettes.

### ENDS’ Functional Role

To varying degrees, ENDS simulated physical smoking attributes, thus supporting transitions from cigarettes: *“. . .* the best thing about it was that it mimicked a cigarette quite well in terms of the smoke, uh, vapour that would come out*”* (Hugh). Some valued the similar *“*feeling of inhaling*”* (Sam) while others valued similar hand actions, which they associated with relaxation: *“. .* . it provided, not the same, but a similar feeling of relaxation and habit. Like, a nice habit of bringing your hand up to your face.” (Jeff)

Participants’ prior cessation attempts and methods varied; ENDS’ similarity to aspects of smoking meant some found ENDS less cognitively effortful than other cessation methods. Pippa explained that “. . . it’s not like a [NRT] patch where I’m just sitting there and I have to just keep conning myself into ‘You are fine!’ . . . I’ve got something in my hand, where it’s a little bit similar, and I might have more chance of conning myself into believing that it’s enough.” Mikaere also felt using ENDS for cessation was “easier” than previous attempts: “Cause with the Alan Carr book, I had to keep getting all like in my head and philosophical . . .”.

Yet although ENDS reduced the effort required to stop smoking, they did not eliminate that effort entirely, and many participants continued to experience strong urges to smoke. Several used self-talk to manage these situations, as they had done in previous quit attempts, as Sam described: *“.* . . it felt like picking up a new routine and being like okay, try to tell yourself, ‘So, I don’t do that now. I do this’ . . . but still thinking about having a cigarette, or smelling a cigarette, or seeing other people smoking, and realise that I could just have one, I could just ask for one and, there’s nothing, there’s really no barrier there . . . like a paper-thin wall between you and a cigarette, and there’s nothing stopping you from doing it, except for you trying to rationalise with yourself.” Sarah explained how ENDS did not completely eliminate cigarette cravings but instead distracted her and “subdued” cravings:“. . . it didn’t completely get rid of the feeling of wanting to have a cigarette, but it gave enough of a distraction to kind of ignore it. It made it feel probably like 90% go away.” Like other participants, previous “cold turkey” quit attempts had caused irritability: “the cold turkey thing just like . . . it had just made me really snappy towards people, because all I wanted was a cigarette and there was nothing else kind of helping me. Whereas the vape just kind of subdued it in a way” (Sarah).

Physical similarities allowed some participants to maintain familiar smoking and socializing practices, as Anita explained:

*“...so when it came to night-time it wasn’t just a blank space of ‘what do I do now?’… I could kind of just go about the exact same routine and watch the telly or drink a juice or something on the deck and vape instead of smoking, rather than not have anything at all”.*

Similarly, Natalie found quitting with ENDS easier than with NRT patches, as she found ENDS use interchangeable with smoking in social situations:

*“I think if you’ve got a routine, like especially when you’re working, you still go outside, and you still do that, and if there’s someone you do it with, you can still have a chat to those people… Whereas, when I was trying with the patches and stuff, it was just- you missed the actual ‘event’, there was nothing. You just kept going.”*

The interchangeability of ENDS and smoking fostered connections with other nicotine users:

*“I think it was important to still fit in somewhere... smoking and vaping gives you that.... and it gives you, I guess, your little village of people who are also doing that… whereas if you don’t smoke or vape, you sort of lose it and you lose those connections”* (Mackenzie).

### ENDS’ Inauthenticity

Despite mimicking some smoking attributes, many participants found ENDS less authentic and satisfying than smoking; Hugh described ENDS as “a substitute . . . not the real thing” (Hugh), and others felt they were “missing something” (Natalie). Participants’ experiences varied; some found the flavors artificial, others disliked the device mouthpiece, or the aerosol’s mouthfeel, and yet others found the hand-feel less satisfying, as Mikaere explained:

*“Even just flicking the cigarette is satisfying to me because literally when I have a cigarette in my hand that’s what I do the whole time just flicking… I suppose you could develop some habits with- [ENDS], I can’t really think of anything.”*

While Mackenzie felt ENDS (like smoking) supported social connections, others found ENDS did not help social interactions; Callum commented: “smoking actually brought people together; vaping doesn’t do that”. More fundamentally, ENDS did not provide the same comfort, relaxation, or pleasure as smoking:

*“There’s almost a sense of satisfaction from sitting and having a cigarette, whereas when you have the vape, it’s just, the experience is - it’s different. Maybe because the smell’s different and, and the taste’s different. I don’t think I really enjoyed it. Like, I quite enjoy sitting down, having a cuppa and kicking back and having a cigarette with it. Just with the vape I just didn’t find any pleasure in it whatsoever.” (Tabitha)*

Participants described tobacco as “comfortable”, “natural”, “like old times” or “an old friend”, metaphors that revealed long-standing, nostalgic, attachments to smoking; these descriptions contrasted sharply with ENDS, which they described as “foreign”, “odd” or “strange”. ENDS’ inauthenticity was most obvious when participants saw others smoking:

*“…it’s good until I’m around people that are smoking. Then I’m just like, ‘Oh. It’s not the same!’… I don’t know. I feel like there’s just a difference between vaping and smoking.... It’s really hard to explain.” (Leigh)*

Physical attributes, such as the smell of others’ cigarettes or highly paired practices involving smoking and alcohol, attracted some back to smoking: “. . . the hit doesn’t feel the same.... there’s no tickle at the back of the throat which I really like, especially when I’m drinking . . . smoking when you’re drinking, I don’t know, it just feels really good” *(Jeff).*

Stress often contributed to smoking resumption as ENDS did not provide the relief smoking had offered. While participants persevered with ENDS in everyday life, even while preferring cigarettes, some situations spurred a desire for comfort or a “treat” that only cigarettes provided:

*“… cigarettes is always, obviously, the preference. And, you know, the vaping is to try to wean me off it, but they’re still, obviously, that’s very much the preference. So I have weak moments, like if I’ve been drinking, or if it’s been really stressful at work, I might decide that I deserve it* [smoking] *as a treat.” (Pippa)*

### Perpetuating Addiction and Risk of Harm

Several participants reported ENDS offered greater convenience than smoking and felt ENDS enabled short, quick usage sessions that cigarettes did not: “. . . it’s so much easier to just pull it out, have a puff, whack it back in. ‘Cause for having a fag, you need to really commit to finishing the cigarette” (Mikaere). Yet, while the convenience Mikaere described initially appealed to participants, most found this attribute made ENDS use difficult to control and could lead to overconsumption:“. . . it’s way harder to stop yourself simply because you can just have it all the time, pretty much, . . . the simplicity of it and how cheap it is, and easy” (Jake). More frequent ENDS use relative to smoking also reflected different consumption constraints; unlike smoking, which took place outdoors, participants used ENDS indoors, which facilitated “constant” use:

*“…with a cigarette… you gotta go outside, you pull the cigarette out, you gotta light it. Just, you know, there’s steps. Whereas a vape is literally you’re pressing a button and you’re getting an instant-, like, it’s quick. So, and having that there constantly… it was quite bad” (Jeff).*

Unlike cigarettes, ENDS lacked a defined finish, which left some participants uncertain when to conclude a session: “. . . it’s like, ‘When’s it enough?’ . . . you sit there and it’s like, ‘You’ve been sucking on this for five minutes. You know you’ve got the nicotine in you, you should stop now.’” (Pippa). Some participants felt “tied to” (Stacey) their ENDS and described these as a “permanent attachment”:

*“It kind of ends up being with you all the time. Like a permanent attachment… I just sort of carried it around places, whereas at home I don’t carry around a pack of smokes in my hand…” (Natalie)*

The perceived risk of ENDS overuse meant participants felt they needed to self-regulate to avoid increasing their nicotine dependence; Erin explained:“. . . when I quit smoking the second time, I regulated the use of my vape way more than what I did the first time . . . .So I was only using my vape the second time when I actually felt like I needed it. Whereas the first time I was just kind of always puffing on it . . .”. Natalie also found adapting to ENDS required self-regulation; as her consumption became more frequent, it undermined the “treat effect” that smoking had provided:

*“I think it’s a good idea to try and use your vape like a cigarette… even when you’re at home, you know, if you think, “this is when I’d like to have a smoke”, go outside and do that so you keep that ‘treat’ effect, perhaps. That it’s not just something that you’re constantly puffing away on…”*

Because ENDS’ accessibility typically fostered more frequent use, a minority experienced adverse effects that they attributed to overuse. Jeff explained:

*“…mindset wise and physically it was starting to make me feel bad and I think it had a big impact on my ability to sleep and eat..., ‘cause I was vaping all the time, constantly stimulated, and appetite-suppressed sort of thing. I just never felt hungry, never slept and always felt kinda slightly sick.” (Jeff)*

Regardless of whether participants experienced any adverse effects from ENDS, several expressed concern over the uncertain long-term effects of ENDS, as Tabitha explained:

*“…with a cigarette, you know, if you break it down... you know exactly what’s in it, even though they’re all still bad for you. But it’s kind of defined. Whereas with a vape, for me personally, I didn’t know what I was actually putting into my body…. I always felt like, mm. I don’t even know if I wanna be doing this”.*

Participants drew contrasting conclusions about ENDS’ role in helping them switch away from smoking. Several thought ENDS had “created its own problem” (e.g., Jake) and a minority wondered whether perpetuating the “habit” increased the risk of smoking resumption:

*“I found that when you try to quit smoking but replace it with ‘smoking’ essentially* [participant here described vaping as ‘smoking’]*, it’s really difficult to drop that habit when you’re filling it with something else. I don’t know, smoking and vaping are still quite similar and the fact that there is ‘smoke’ and there is still that hand-motion of having a cigarette or a vape… it’s so much easier to fall back to smoking.” (Mackenzie)*

Despite resuming smoking, participants still viewed ENDS as a viable option to help them switch from smoking, though many saw them as an intermediary step in their journey to becoming nicotine-free, rather than as “quitting”: “Quitting for me is using neither. Not having cigarettes or not using the vape” (Warren).

## Discussion

Our themes suggest that participants’ willingness to continue using ENDS eroded, particularly as smoking still attracted them. Although participants gained enjoyable physical sensations from ENDS and found similarities between ENDS and cigarettes, those likenesses did not go far enough; participants sought replication rather than similarity. While ENDS’ similarities to cigarettes facilitated the initial transition from smoking to ENDS use, some found those parallels also served as cues that prompted a return to smoking. Concerns about overusing ENDS, and the unanticipated effort to self-regulate ENDS consumption in addition to resisting smoking urges, also contributed to smoking resumption. During stressful periods, cigarettes’ unique sensory attributes provided familiarity, pleasure, and comfort that participants did not associate with ENDS and, for some, ENDS did not offer the “authentic” social connections smoking provided.

We add to existing evidence regarding the challenges of using ENDS as a reduced harm alternative. Numerous quantitative studies have reported that people attempting to switch to ENDS often find them less satisfying than conventional cigarettes. e.g.,^28, 29^ Considerations of “satisfaction” have often focused on nicotine delivery and ensuring people can address their cravings. For instance, some services simply encourage people to “choose the right strength or nicotine to satisfy your needs”.^30^ Yet participants’ associations with smoking went well beyond accessing nicotine and suggest that satisfaction should be reconceptualized. We extend earlier studies by explaining how lack of satisfaction with ENDS includes devices’ unfamiliarity, physical inauthenticity (e.g., hand action), failure to provide a demarcated pleasurable “treat”, or inability to maintain authentic social connections.^19^ Experiences of these mismatches across personal and social domains provided ongoing reminders that ENDS, while similar to cigarettes, did not replicate smoking. When socializing with people who smoke, drink alcohol, or experiencing stress, the impact of these reminders was particularly apparent and cued a return to smoking.^19,31^

Much of the ENDS-related information in the public domain presents the transition to ENDS as straightforward, with little mention of dissatisfaction or difficulties switching, including smoking resumption, dual-use, or increased nicotine dependence as unintended outcomes.^32–35^ Implying transitions may not require sustained effort could encourage initial switching attempts but inadvertently prevent people from developing the persistence they later need to resist physical and social smoking cues.^36^ Evidence that ENDS use may lead to continued or increasing nicotine dependence,^19,31,36,37^ negative effects such as headaches and nausea,^38^ and lost sensations and associations,^4^ raises the possibility that providing information on these outcomes may prepare people hoping to transition from smoking to ENDS rather than deter them.

Our findings suggest that advising people of the challenges involved in switching from smoking to exclusive ENDS use, including recommending they monitor their ENDS use and treat vaping like smoking (i.e., a discrete rather than continuous activity), could improve their ability to manage smoking triggers. For example, encouraging ENDS use outdoors only could provide a structure some participants found difficult to self-impose and may help prevent the constant use they found unsettling. Taking specific ENDS-breaks could feel more satisfying than continual grazing and provide a defined “*treat*” that people who smoke value. Advice such as this could be integrated into mass media communications disseminated via health agencies (e.g., ^30^), included in or on ENDS product packaging, or communicated via health professionals in jurisdictions where smoking cessation services support people transitioning to ENDS from smoking.

Encouraging ENDS users to replicate these smoking practices would also protect others in indoor settings from exposure to secondhand aerosol, which may contain harmful chemicals.^39^ However, treating ENDS use and smoking as similar could increase ENDS users’ exposure to smoking; measures to reduce exposure to smoking in public settings thus remain crucial.^40^ Specific examples could include eliminating smoking areas outside bars and introducing smoke-free city centers,^41^, ^42^ while allowing ENDS use in designated exterior spaces.

Like all studies, ours has limitations. We recruited a diverse rather than representative sample, though diversity is typically regarded as a strength of qualitative work.^21^ Our sample comprised people who smoked and had not made a sustained transition to exclusive ENDS use; it thus represents only a specific subgroup of ENDS users. Although we collaborated with Māori researchers, our work is not a kaupapa Māori study, nor was it Māori-led; instead, we aimed to include Māori participants, but not to derive culturally specific data. We compared themes identified independently by a Māori researcher with those we had tentatively proposed and found strong overlap (e.g., the role of smoking and vaping in socializing and dissatisfaction with ENDS); we thus analyzed the data collectively but recognize that, with more Māori participants, we may have identified differences between Māori and non-Māori. Future work using a kaupapa Māori approach and/or led by Māori researchers, and involving a larger number of Māori participants, would help identify factors contributing to smoking resumption that are specific to Māori who smoke. Such research would also offer richer insights into whether reduced-harm nicotine products could potentially benefit Māori, which would have important equity implications.

Despite these limitations, our findings provide rich insights into how smoking resumption after ENDS use occurs in people’s everyday lives. In particular, they suggest that reconceptualizing satisfaction to encompass psychosocial, as well as physical factors, could more effectively recognize the many triggers people attempting to switch must manage. Further work could develop and test self-management tools that could foster transition from smoking to ENDS. In the interim, continuing to regulate smoking could reduce challenges people switching from smoking to ENDS use must manage.

## Supplementary Material

ntac157_suppl_Supplementary_taxonomy_form

ntac157_suppl_Supplementary_File_S1

## Data Availability

Interview transcripts cannot be shared publicly because of ethical restrictions. However, excerpts of the transcripts can be made available, via a supplementary codebook, upon reasonable request.
