# Supplementary material for: A Qualitative Analysis of the Experiences of People Who Resumed Smoking Following Exclusive Electronic Nicotine Delivery Systems Use
Source: Nicotine Tob Res. 2022 Nov 30;25(3):470–7. doi: 10.1093/ntr/ntac157 (PMC9910152; doi:10.1093/ntr/ntac157)
Supplement: ntac157_suppl_Supplementary_File_S1 [file ntac157_suppl_supplementary_file_s1.docx]

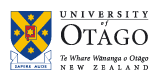


**Vaping to Stay Smokefree**

**Interview Guide**

**Introduction *(5 – 10 mins)***

Kia ora/ hello – I’ve arranged to talk to you about some research I’m doing into smoking and relapse.

Before we start I’d like to show you some information about the session and check to see whether you have any questions about my work. Here’s another copy of the information sheet you were sent when you indicated you were interested in the project; please take a few moments to look through this sheet.

- **Provide participants with an Information Sheet and relevant Consent Form and outline the key points in this.** **ALLOW TIME TO READ THE INFORMATION SHEET.**

Do you have any questions about the study?

- **Explain recording of the interview and participants’ rights in relation to these recordings.**
- **ONCE PARTICIPANTS HAVE AGREED TO INTERVIEW BEING RECORDED, TURN RECORDER ON AND NOTE THAT IT IS NOW ON.**

**READ OUT WHILE RECORDER IS ON:**

As participants in the research, you have the right to ask questions at any time, to decide if you would prefer not to answer some questions, to receive a copy of the findings, and to withdraw from the study at any time up to the end of this interview with no penalty to yourself.

- **Check again whether PARTICIPANTS have any questions about the interview.**
- **If no questions, ask the participant(s) to sign and date the Consent Form**
- **Give participant koha and obtain signed koha forms**

Please note that your responses will be confidential to the research team members. We will assign you a pseudonym, a fake name that is different to your real name, and that name is what you will be known as; if we report any quotes from this interview, they will be attributed to that name and we will not use any person’s real name.

Please also note that we use an online service called Rev.com to listen to the audio files and type up our conversation word-for-word. Rev.com transcribers are located around the world and we do not know who will transcribe the audio file from this interview. There is a very small possibility that someone could transcribe the file and recognise your voices.

**ARE YOU HAPPY FOR ME TO START THE INTERVIEW NOW? CHECK PARTICIPANTS HAS HAD THEIR QUESTIONS ANSWERED.**

**A. Introductory questions (5 mins for A and B)**

- When did you start smoking?
- And when did you start vaping? What led you to start vaping?
- When you started vaping, what did you want to happen with your smoking? And what did happen with your smoking after you began vaping? How did vaping fit into your daily life?
- To what extent were you using a vape to try and quit smoking? (could probe here – How strong were your intentions to quit smoking when you started using a vape? What were the main reasons for using a vape?)

**B. Tell us about your experience of being smokefree & relapsing**

- As you know we’re interviewing people who have stopped smoking for a while by using an e-cigarette or a vape, but then have gone back to smoking. How many experiences like that have you had?
- Perhaps you can talk us through that experience/ your most recent experience?

(Depending on response, can use relapse sections as initial probes,

then refer back to the initiating vaping sections)

**C. Initiating vaping for quitting – 5 mins ***Ask these questions only of ‘deliberate’ quitters***

- Before you tried vaping, had you tried to quit smoking before? Could you tell us how you tried to quit? And how did your quit attempt go?
- Thinking now about when you used a vape to quit, **what** got you thinking about giving up smoking?

(probe – external ‘forced’ attempt, or internal motivation, or mixed)

- **What** led you to use a **vape** in your quit attempt?
- **What** did you think it would be like trying to quit smoking using a vape? How did your expectations compare to actually using the vape?
- **What** sort of preparation did you do, going into the quit attempt? (probe – did you seek any support or advice? If yes, what sort of support/ advice, and from who? How useful was that support/ advice?)
- How **confident** were you feeling about being able to stay smokefree? (probe – how did vaping affect your confidence levels? How did those feelings evolve over time?)
- Compared to earlier quit attempts when you didn’t use a vape, how did you find trying to quit using a vape?

**D. Initial transition to vaping from smoking – 5 mins ***Ask these questions of all participants***

- What type of vape did you use? How did you decide on that model? How did you find using it? What level of nicotine did you use? (probe – changes in nicotine level over time) Which flavours did you use?
- How soon did you start vaping regularly? What happened to your smoking? (Probe - did you reduce your cigarettes over time or switch from smoking to vaping in one go?)
- How did you find the first week after you had stopped smoking?
- What sort of feelings or symptoms did you experience in the first week or so after you stopped smoking? (Probe - feeling irritable, anxious, feeling down or low, trouble sleeping). How did you manage those? (probe – role of vape)
- How often did you experience **urges to smoke**? What were those urges like? (probe – whether a constant background craving, or more sudden, intense urges?) How did you manage those? (probe – role of vape)

**E. Vaping establishment – 5 mins**

- As time went on, how did you find replacing smoking with vaping? (Probe: What similarities and differences did you notice? How did you manage these?)
- How much control did you feel you had over vaping compared to smoking?
- How did you feel about yourself once you had stopped smoking?
- How easy was it to connect with friends and family once you had stopped smoking?
- What reactions did you get from friends and family when you stopped smoking?
- When you’d stopped smoking, what did you think would happen with your vaping? (Probe: Did you plan to quit vaping or use the vape long-term? What happened? (If tried and failed to quit ENDS: Do you think that influenced your move back to smoking? What makes you say that?)

**MAIN SECTIONS**

**F. Lapses and progression from lapsing to relapse – 10 mins**

- - Were there any times after you’d stopped smoking when you smoked again, even just a puff of a cigarette?

(If yes, can you tell me what happened? How soon after stopping smoking did you have a puff? Whereabouts were you? What do you think led you to have a puff again? (**Probe:**  Was it something you planned or did it just happen? Was it triggered by something internal, or something external? Or was there something wrong with your vape?)

- How did you feel afterwards about having a puff? (**Probe:** How did having a puff make you feel about quitting?)
- What impact do you think it had on your quit attempt? (or for accidental quitters – on their smoking)
- What did you do to try and stay smokefree after having had a puff? How well did that work? (Probe: How did you use your vape to stay smokefree after the lapse? Were there any things you did differently with vaping after having had a puff again? What were these changes and how did they work out?)
- What happened after you first had a puff on a cigarette again? (**Probe:** did you stop smoking again or did you start having more puffs on a cigarette?)
- [If relevant] Thinking back to your earlier quit attempts when you *didn’t use* a vape, what happened after you had had a puff on a cigarette?

**G. Relapse: settings, triggers & contributors – 10 mins**

Now I’d like you to think about how you took up smoking more regularly again, and what happened.

- At what stage did you start smoking regularly again? (Probe – how long after the quit attempt/ puff on a cigarette?) What led you back to smoking, do you think? (Probe – side effects – explore what these were. Lack of satisfaction? Difficulty connecting with others? Stigma of vaping? Effects of vaping on health?)
- Are there any other things that led you back to smoking?
- What were you looking for when you moved back to smoking? What did you get from smoking that you didn’t get from vaping?
- How did your thoughts or feelings about smoking affect your move back to smoking? What about your thoughts or feelings about vapes, how did these affect moving back to smoking?
- Overall what impact do you think vaping had on becoming smokefree? What about on returning to smoking?

**H. Reactions to relapse – 10 mins**

- How did you feel about moving back to smoking? (probe – disappointment or relief? Was there anything positive you gained? If yes, what?)
- How have those feelings evolved over time?
- What do the important people in your life think and feel about your move back to smoking? How has that affected your smoking?

**FINAL SECTION**

**I. Future intentions- 5 mins**

- - What role does vaping have for you now?
  - What are your future intentions with smoking? And how about for vaping?
  - Do you plan to stop/ quit again using vaping?
  - **What would you do differently in your next quit attempt? (**or for accidental quitters ‘next time you stop smoking’) (Probe – different device, strategy/ preparation? settings management?)
  - **What do you think would have helped you stay smokefree?
  - **Do you have any specific advice about triggers and how these could be managed to avoid relapse?

**Conclusion**

These are all the questions I wanted to ask you. Do you have any other comments you’d like to make?

Finally, I just have a short questionnaire for you to complete. Like the rest of our discussion, the information you provide will be completely confidential and only members of the research team will be able to access it.

**PROVIDE RESPONDENTS WITH BACKGROUND QUESTIONNAIRE AND COLLECT AND CHECK ON COMPLETION**

**Ensure each participant who has received a $40 koha has signed the gift voucher receipt**
